# Composite Material Formation Based on Biochar and Nickel (II)-Copper (II) Ferrites

**DOI:** 10.3390/molecules30193900

**Published:** 2025-09-26

**Authors:** Nina P. Shabelskaya, Alexandr V. Vyaltsev, Neonilla G. Sundukova, Vera A. Baranova, Sergej I. Sulima, Elena V. Sulima, Yulia A. Gaidukova, Asatullo M. Radzhbov, Elena V. Vasileva, Elena A. Yakovenko

**Affiliations:** 1 Department of Ecology and Industrial Safety, Faculty of Technology, Platov South-Russian State Polytechnic University (NPI), 346428 Novocherkassk, Russia; bgd-av@mail.ru (A.V.V.); sundukovang@serconsrus.com (N.G.S.); u.vera20@yandex.ru (V.A.B.); yu-npi@yandex.ru (Y.A.G.); rajabov.asadullo@mail.ru (A.M.R.); karalenka5@yandex.ru (E.V.V.); yakovlena80@yandex.ru (E.A.Y.); 2 Laboratory of Agrobiotechnology for Improving Soil Fertility and Agricultural Product Quality, Southern Federal University, 344006 Rostov-on-Don, Russia; 3 Department of Chemical Technologies, Faculty of Technology, Platov South-Russian State Polytechnic University (NPI), 346428 Novocherkassk, Russia; s_sulima@mail.ru (S.I.S.); elena-sulima66@mail.ru (E.V.S.)

**Keywords:** copper (II) ferrite, nickel (II) ferrite, Jahn–Teller effect, organic dye removal, water purification

## Abstract

This paper studies the formation process of a composite material based on an organic substance, biochar from sunflower husks, and an inorganic substance, nickel (II)-copper (II) ferrites of the composition Cu_x_Ni_1−x_Fe_2_O_4_ (x = 0.0; 0.5; 1.0). The obtained materials were characterized by X-ray phase analysis, scanning electron microscopy, and FTIR spectroscopy. It is shown that when replacing copper (II) cations with nickel (II) cations, the average parameters and volume of the unit cell gradually decrease, and the cation–anion distances in both the tetrahedral and octahedral spinel grids also decrease with regularity. The oxide materials were found to form a film on the surface of biochar, repeating its porous structure. The obtained materials exhibit high catalytic activity in the methyl orange decomposition reaction under the action of hydrogen peroxide in an acidic medium; the degradation of methyl orange in an aqueous solution occurs 30 min after the start of the reaction. This result may be associated with the formation of the Fenton system during the oxidation–reduction process. A significant increase in the reaction rate in the system containing mixed nickel–copper ferrite as a catalyst may be associated with the formation of a more defective structure due to the Jahn–Teller effect manifestation, which creates additional active centers on the catalyst surface.

## 1. Introduction

Nowadays, the expansion of industrial production has led to a continuous increase in environmental pollution. According to [1], more than 100,000 tons of synthetic dyes are produced each year, with azo dyes (compounds containing the bond –N=N–) accounting for almost 70% of the total volume of dye production [2]. The ingress of dyes into natural water bodies leads to a decrease in the flow of light, which is accompanied by a negative impact on aquatic and soil ecosystems, and interferes with the process of photosynthesis [3]. In addition, the toxic and carcinogenic properties of azo dyes pose a danger not only to humans but also to other living beings [3]. In this regard, an urgent task is to find effective methods for cleaning water resources from pollutants.

One of the promising methods of protecting water from dyes is purification using adsorption or oxidative destruction processes. Transition metal ferrites with a spinel structure are promising materials in many applications due to the successful combination of several properties, such as magnetic and dielectric.

Ferrites are a special type of ferrimagnetic material [4], which contains a grid of oxygen anions and cations of three- and two-charged metals distributed in it. The spinel formula can be represented as A^2+^B_2_^3+^O_4_, where A and B represent metal cations. Depending on several factors, the spinel structure can be “normal” (all A^2+^ cations are located in tetrahedral grid positions, or A-positions, all B^3+^ cations are located in the centers of octahedra, or B-positions), “inverted” (all A^2+^ cations and half of the B^3+^ cations occupy octahedral sites, half of the B^3+^ cations are located in tetrahedral voids) [5,6].

However, in nature, spinels are encountered most often with a partially inverted structure. Replacing the A^2+^ metal in the spinel composition can lead to a redistribution of A^2+^ and B^3+^ ions over the crystal grid nodes and, hence, to the appearance of new favorable properties [7]. In addition, several such compounds can experience crystal grid distortion due to the Jahn–Teller effect manifestation [8,9,10,11]. In this case, the crystal symmetry decreases, and a transition from the cubic (*Fd*3*m*) to the tetragonally distorted (*I*4_1_/*amd*) phase occurs [12]. For example, copper (II) ferrite can exist in a cubic [13] or tetragonal [12] modification. Formation of areas with a defective structure can have a positive effect on the spinel’s catalytic and adsorption properties.

Spinel ferrites, due to their unique properties, have numerous potential applications in various industries, for example, in medicine for targeted drug delivery [14], as sensors for detecting hazardous substances [15,16,17], and in the processes of heat [18] and electric energy [19,20,21] transfer and accumulation. In recent years, ferrites have been successfully used as inexpensive, effective catalysts for hydrogen production [22], cyclic hydrocarbon oxidation [23], and dye decomposition [24,25,26,27].

Table 1 shows some data on the degradation of the methyl orange dye by the action of hydrogen peroxide in the presence of a catalyst.

The properties of ferrites with a spinel structure are significantly affected by the composition, production processes, and heat treatment mode. To obtain ferrites, both traditional methods, such as ceramic [36], coprecipitation method [37,38,39], and relatively new ones, such as hydrothermal [40,41], and sol–gel technology [4,25,26,42] are used.

Synthesis of organo-inorganic composite materials can obtain compounds with new properties characteristic of individual substances included in the composite [17,41]. Taking into account the above, the current work aimed to obtain composite materials based on an organic substance, biochar from sunflower husks, and an inorganic substance, nickel(II)-copper(II) ferrites of the composition of Cu_x_Ni_1−x_Fe_2_O_4_, (x = 0.0; 0.5; 1.0), and to study their catalytic activity in the azo dye—methyl orange—decomposition reaction.

## 2. Results and Discussion

During the synthesis process, the formation of a porous gel-like substance was observed first, then its combustion occurred, and, ultimately, the samples became black powders. The resulting composite materials were studied using the X-ray phase analysis method.

**Figure 1:**
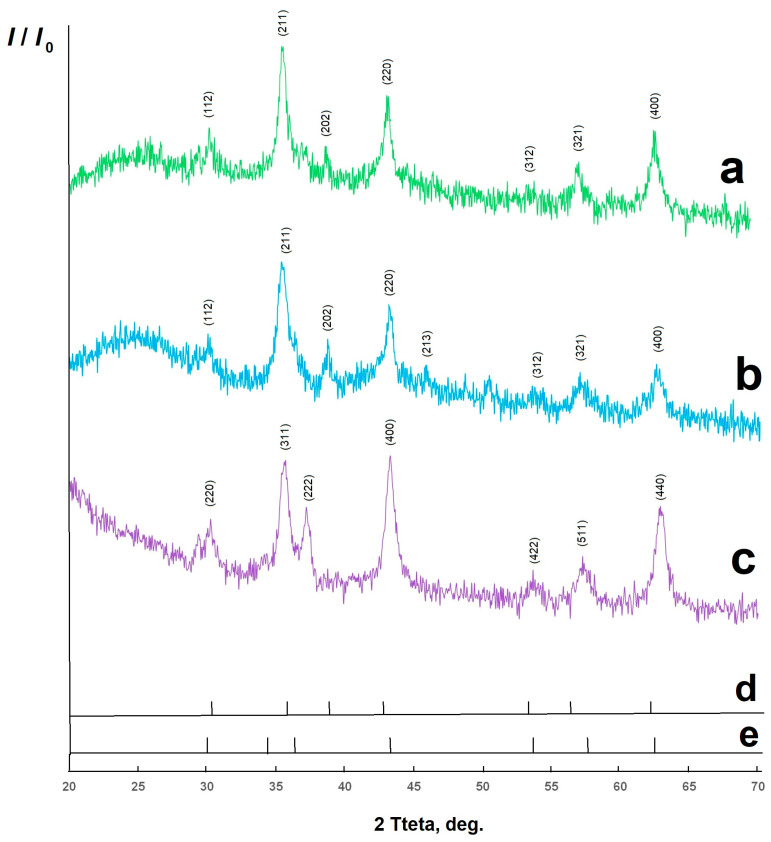
X-ray diffraction pattern of CuFe_2_O_4_/biochar composite materials (a), Cu_0.5_Ni_0.5_Fe_2_O_4_/biochar (b), NiFe_2_O_4_/biochar (c), reference peaks for NiFe_2_O_4_ (d) and CuFe_2_O_4_ (e).

Figure 1 shows the X-ray patterns of the synthesized materials.

The organic part of the composite material is X-ray amorphous. The inorganic part is copper (II) ferrite CuFe_2_O_4_, PDF Number 000-34-0425, tetragonal modification *I*4_1_/*amd* (Figure 1 (a)), nickel (II) NiFe_2_O_4_, PDF Number 000-54-0964, cubic modification *F*d3*m* (Figure 1 (c)), mixed nickel (II)-copper (II) ferrite (Figure 1 (b)). NiFe_2_O_4_ is characterized by peaks at the angles (2θ): 30.3; 35.7; 37.2; 43.3; 53.8; 57.35; 63.0, for which the parameter values (h, k, l) are respectively set to 220; 311; 222; 440; 422; 511; 440. CuFe_2_O_4_ is characterized by peaks at the angles (2θ): 30.32; 35.64; 38.88; 43.48; 53.84; 57.2; 63.0, for which the parameter values (h, k, l) are respectively set to 112; 211; 202; 220; 213; 312; 321; 400. Cu_0.5_Ni_0.5_Fe_2_O_4_ is characterized by peaks at the angles (2θ): 30.24; 35.56; 38.8; 43.24; 53.76; 56.92; 62.76, for which the parameter values (h, k, l) are respectively set to 112; 211; 202; 220; 213; 312; 321; 400. It is interesting to note that the 2θ angle values for mixed nickel (II)-copper (II) ferrite are not between those of pure nickel (II) ferrite and copper (II) ferrite but are usually smaller. This may be due to the greater defectiveness of the inorganic material being formed.

Table 2 presents data on the calculations of the unit cell parameters of ferrites (a, c), nm; the degree of tetragonality (c/a); the average cell parameter *a_m_*, nm; the unit cell volume *V*, (nm)^3^; the crystallite size *D*, nm; the “anion-cation” distances (nm) in the tetrahedral (*L*_A_) and octahedral (*L*_B_) coordination of the spinel grid.

For the synthesized spinels in the composite material, the inversion parameter λ, grid deformation (ε), dislocation density (δ), and X-ray density (ρ) were calculated. The results are presented in Table 3. When compiling the chemical formulas, it was considered that nickel (II) and copper (II) cations tend to be located in the B-positions of the spinel structure [12], and for Ni^2+^ this preference is more pronounced. Fe^3+^ cations do not have a pronounced predisposition to occupy tetragonal or octahedral voids.

The obtained X-ray phase data analysis results show that when replacing copper (II) cations with nickel (II) cations, which have a smaller size (the radii of these cations are 0.080 and 0.074 nm, respectively), the average parameter and the volume of the unit cell gradually decrease. The cation–anion distances in both the tetrahedral and octahedral spinel grids also decrease with regularity.

**Figure 2:**
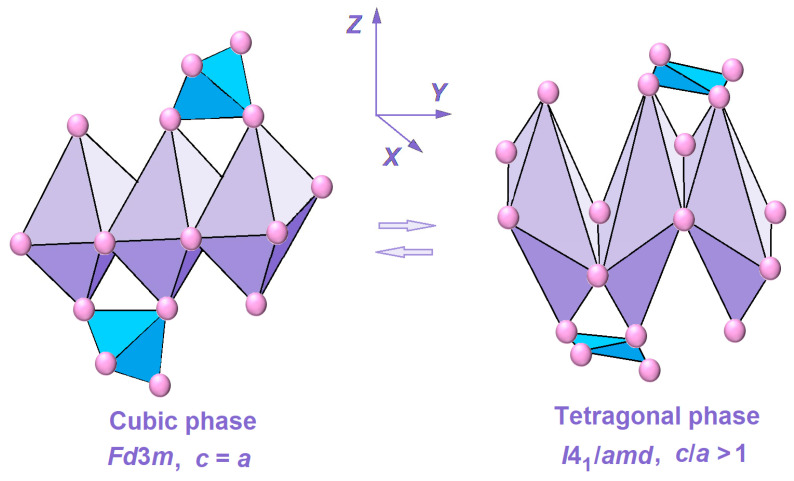
Schematic representation of the Jahn–Teller effect manifestation during the formation of CuFe_2_O_4_ tetragonal phase.

However, an anomaly in the value of the grid parameter of a for mixed nickel–copper ferrite should be noted: it was less than the expected value. This experimental fact can be associated with a high degree of nickel-containing ferrite inversion and the formation of a more defective spinel structure due to the Jahn–Teller effect manifestation. In the latter case, the symmetry of the oxygen framework decreases, and areas with octahedra elongated along the z axis are formed. The formation of a distorted spinel structure under the influence of the Jahn–Teller effect is schematically shown in Figure 2.

The formation of a defect structure in mixed nickel–copper ferrite is accompanied by an increase in the X-ray density value (Table 3). For the same sample, the grid deformation and dislocation density values are 32.5 % and 18 % higher, respectively, compared to these values for NiFe_2_O_4_.

**Figure 3:**
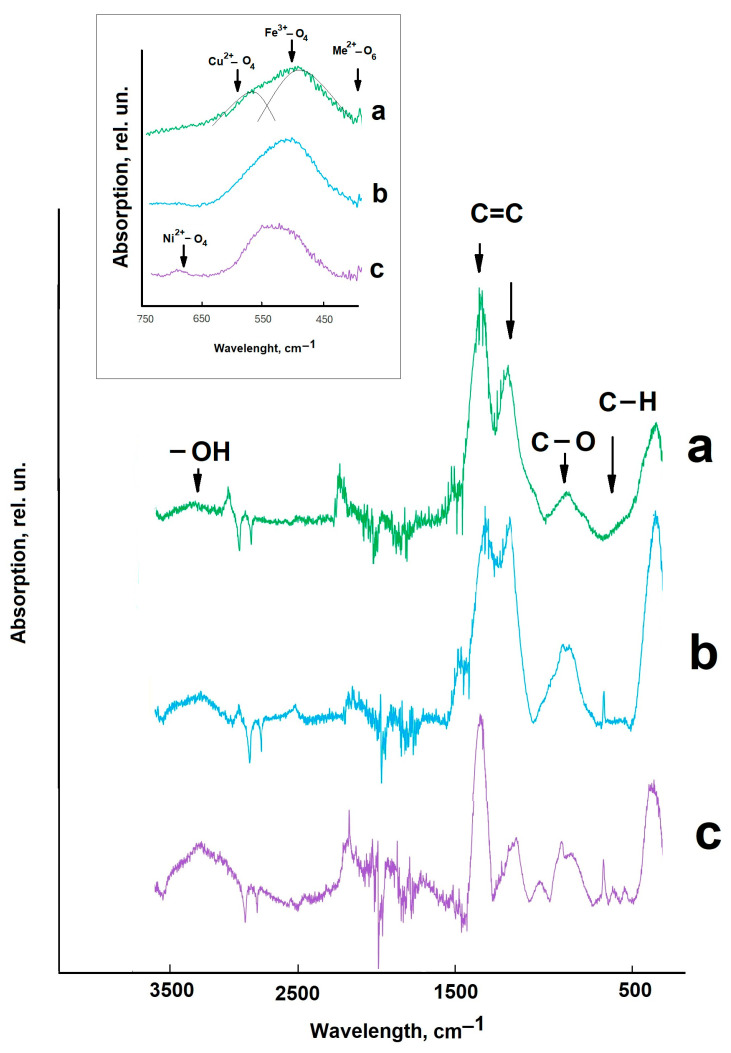
FTIR spectra of synthesized composite materials: CuFe_2_O_4_/biochar (a), Cu_0.5_Ni_0.5_Fe_2_O_4_/biochar (b), NiFe_2_O_4_/biochar (c).

The FTIR spectroscopy data complement the results of the structural features analysis. Figure 3 shows the FTIR spectra of the synthesized composite materials; the recording was carried out in the wavelength range of 500–3500 cm^−1^.

In the range of 800–3500 cm^−1^, oscillations of the composite material organic component appear [17]. Peaks in the range of 3000–3500 cm^−1^ are associated with vibrations of -OH groups [17]. In the range of 1500–1700 cm^−1^, intense peaks of C=C bonds appear. The peaks in the range of 1000–1200 cm^−1^ and 800 cm^−1^ are associated with vibrations of C-O and C-H groups, respectively [43]. In the range of 500–800 cm^−1^, vibrations of the composite material oxide component appear [17,40] (in Figure 3, this region is highlighted in the insert). In this case, vibrations of the bivalent metal (Ni^2+^ or Cu^2+^) in the octahedral positions of the spinel grid are distinguished in the region of 450 cm^−1^.

The broad peak in the region of 550–600 cm^−1^ is associated with vibrations of Fe^3+^ in tetrahedral positions. In the region of the largest wavelengths of 650–750 cm^−1^, peaks of vibrations of two valence cations (Ni^2+^ or Cu^2+^) in tetrahedral coordination appear. It should be noted that for copper-containing composite materials, the broad peak in the region of 500–700 cm^−1^ is not symmetrical; it can be decomposed into several components, unlike the composite with NiFe_2_O_4_ (Figure 3, insert). Thus, the FTIR spectroscopy data also indicate the formation of a structure with increased defectiveness in mixed nickel–copper ferrite.

**Figure 4:**
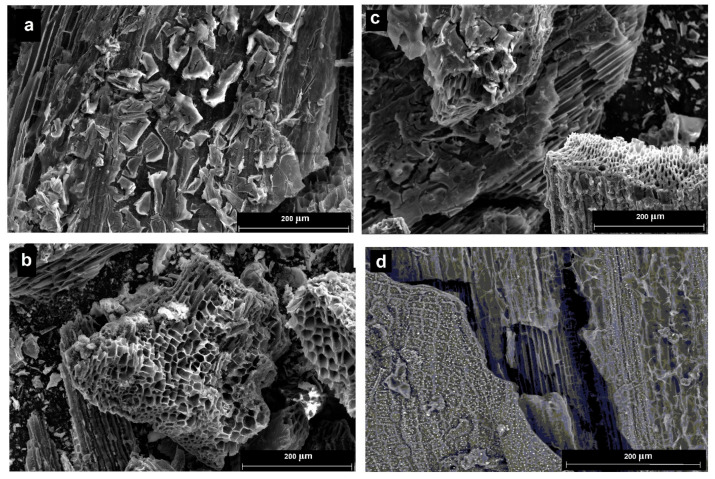
SEM images of the synthesized materials: CuFe_2_O_4_/biochar (**a**), Cu_0.5_Ni_0.5_Fe_2_O_4_/biochar (**b**), NiFe_2_O_4_/biochar (**c**), biochar from sunflower husk (**d**).

Figure 4 shows micrographs of the synthesized materials (a–c) and pure biochar (d).

Evidently, the oxide materials form a film on the biochar surface, repeating its porous structure. This can be useful for the catalytic properties of the materials, as materials with a developed surface are formed, which is important for catalysts. However, the value of the specific surface area decreases by about 20% (for the composite Cu_0.5_Ni_0.5_Fe_2_O_4_/biochar, it was 42.3 m^2^/g).

The synthesized materials were tested in the azo dye degradation reaction under hydrogen peroxide in an aqueous solution. To study the effect of the medium acidity on the destruction process course, the catalytic properties of the materials were studied at pH values of 1, 6, and 11. For this, 0.1 mL of sulfuric acid solution or 0.1 mL of sodium hydroxide solution with a concentration of 1 mol/L were introduced into the reaction system. If the process was carried out without introducing additional reagents, the pH of the solution was slightly acidic due to partial dissociation of hydrogen peroxide according to Reaction (1):

H_2_O_2_ = H^+^ + HO_2_^−^. (1)

**Figure 5:**
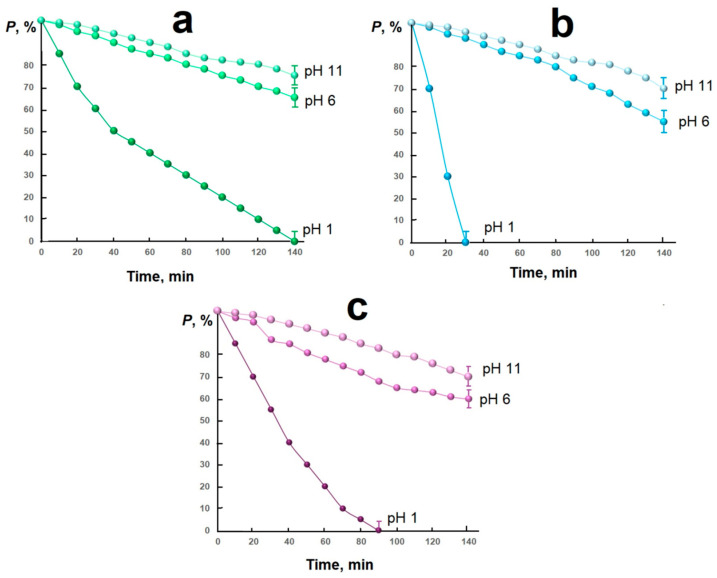
Destruction time of organic dye in aqueous solution in the presence of catalyst: CuFe_2_O_4_/biochar (**a**), Cu_0.5_Ni_0.5_Fe_2_O_4_/biochar (**b**), NiFe_2_O_4_/biochar (**c**).

It was found that in a neutral and alkaline environment, it is not possible to completely purify the aqueous solution from the azo dye: even after 12 h, the concentration of methyl orange in all the conditions studied did not decrease below 50% (mass). The results of purifying the aqueous solution in an acidic environment are shown in Figure 5.

It was determined that the mixed nickel–copper ferrite exhibits exceptional activity in the methyl orange (MO, C_14_H_14_N_3_O_3_SNa) degradation reaction in an aqueous solution (see Table 1): complete destruction of the dye occurs 30 min after the start of the reaction. This result may be associated with the Fenton system formation during the oxidation–reduction process (Equations (2)–(6)):

CuFe_2_O_4_ + H_2_O_2_ → (CuFe^+2^Fe^+3^O_4_)^−^ + O^0^ + H_2_O^+^, (2)

H_2_O^+^ →2H^+^ + O^0^, (3)

2H^+^ + O^0^ = H_2_O, (4)

2(CuFe^+2^Fe^+3^O_4_)^−^ + O^0^ + 2H^+^ → 2CuFe_2_O_4_ + H_2_O, (5)

O^0^ + MO → (NOx + CO_2_ + H_2_O + …). (6)

**Figure 6:**
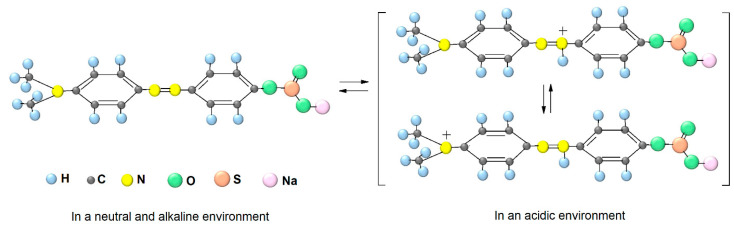
Forms of methyl orange indicator in aqueous solution.

A more complete process of dye degradation in an acidic medium may be associated with an intermediate product formation based on copper (II) ferrite, which has a negative charge. Considering that in an acidic medium methyl orange exists in two modifications with an excess positive charge (Figure 6), it can be assumed that the presence of positive (azo dye) and negative (copper ferrite) particles in the system leads to the dye molecules’ fixation on the catalyst surface and the organic substance oxidation process facilitation by the released active oxidizing particle.

A significant increase in the reaction rate in the system containing mixed nickel–copper ferrite as a catalyst may be due to the formation of a more defective structure due to the Jahn–Teller effect manifestation, which creates additional active centers on the catalyst surface.

Without significant loss of activity, the catalysts can be used for five cycles.

## 3. Materials and Methods

### 3.1. Materials

The composite materials considered in this study are based on an inorganic component, copper (II)-nickel (II) ferrites of the general composition of Cu_x_Ni_1−x_Fe_2_O_4_, (x = 0.0; 0.5; 1.0), and an organic component, biochar from sunflower husk. The materials were obtained in powder form using sol–gel synthesis. For this purpose, the following raw materials were used: aqueous ammonia solution (25 %) (Rushim, Yekaterinburg, Russia), citric acid monohydrate (C_6_H_8_O_7_·H_2_O) (Russian Product, Moscow, Russia), copper (II) nitrate hexahydrate (Cu(NO)_3_)_2_ 6H_2_O) (Rushim, Yekaterinburg, Russia), nickel (II) nitrate hexahydrate (Ni(NO)_3_)_2_ 6H_2_O) (Rushim, Yekaterinburg, Russia), and iron nitrate octahydrate (Fe(NO)_3_)_3_ 9H_2_O) (Rushim, Yekaterinburg, Russia) of chemically pure grade.

### 3.2. Synthesis of Biochar

The synthesis of biochar is described in detail in [44]. The biomass of sunflower husks (Troika LLC, Batrak village, Russia) was thoroughly washed with distilled water, dried until weight loss ceased, and subjected to stepwise pyrolysis without air access at temperatures of 100–700 °C, with a temperature change step of 200 °C, and with a 20 min hold in intermediate phases and a 45 min hold in the final phase. The rate of temperature increase was 11 °C/min. The biochar had a surface area measured by the BET method of 54.6 m^2^/g.

### 3.3. Synthesis of Composite Material

Initially, solutions of transition element salts with a concentration of 1 mol/L and a citric acid solution with a concentration of 6.25 mol/L were prepared; the ammonia solution was used without dilution. Subsequently, 25 g of sunflower husk biochar was measured out and mixed with solutions of transition element salts under continuous stirring. The salt solutions were used in the ratio of (Cu (II) and/or Ni (II): Fe (III)) = (1:2), and 50 mL of iron (III) nitrate solution was used. Then, ammonia (15 mL) and citric acid (25 mL) solutions were added. The mixture was heated to ignition (approximately 600 °C). After the sol converted to gel, it ignited, and a fine powder was formed as a result. The synthesis products were allowed to cool to room temperature.

The stoichiometric equations for the formation of the mixture are as follows (7)

0.5 Cu(NO_3_)_2_ + 0.5 Cu(NO_3_)_2_ + 2 Fe(NO_3_)_3_ + NH_4_OH + C_6_H_8_O_7_ → → Cu_0.5_Ni_0.5_Fe_2_O_4_ + by-products (NOx, CO_2_, H_2_O). (7)

Samples of the composition CuFe_2_O_4_/biochar (indicated as CF), Cu_0.5_Ni_0.5_Fe_2_O_4_/biochar (indicated as CNF), and NiFe_2_O_4_/biochar (indicated as NF) were obtained.

### 3.4. Characterization

To characterize the obtained composite materials, various methods were used, including X-ray diffraction (XRD), transmission electron microscopy, the Scherrer method, and FTIR spectroscopy.

The phase composition was studied on an ARL X’TRA X-ray diffractometer (Thermo Fisher Scientific (Ecublens) SARL, Ecublens, Switzerland) (monochromatic Cu-Kα radiation was used) by the point scanning method (0.01° step, 2 s accumulation time at a point) in the range of 2θ values from 20° to 70°. The crystallite size was calculated using the Scherrer Equation (8) [45]

*D* = 0.94∙λ/(β∙cosθ), (8)

where D is the average crystal size, nm; λ is the X-ray wavelength, nm; β is the peak line width at half its height, rad.; and cos θ is the cosine of the angle for the peak.

The calculated value of the unit cell parameter was determined by Formula (9) for the cubic phase of spinel and Formula (10) for the tetragonally distorted phase of spinel:

1/*d*^2^ = (*h*^2^ + *k*^2^ + *l*^2^)/*a*^2^, (9)

1/*d*^2^ = (*h*^2^ + *k*^2^)/*a*^2^ + *l*^2^/*c*^2^, (10)

where *a*, *c* are the unit cell parameters of the spinel structure, nm; *h*, *k*, *l* are the Miller indices; *d* is the interplanar distance, nm.

The unit cell volume (V, nm^3^) was calculated by Formula (11) for the cubic phase and (12) for the tetragonal phase of spinel:

*V*_k_ = *a*^2^, (11)

*V*_t_ = *a*^2^·*c*. (12)

The average unit cell parameter for the tetragonally distorted phase of spinel was determined by Formula (13):

(13) $$a_{m}=\sqrt[{3}]{a^{2}\cdot c}.$$

Grid deformation (ε), dislocation density (δ), and X-ray density (ρ) were determined using Formulas (14)–(16) [40]:

ε = β/(4·tan θ), (14)

δ = (15 · ε)/(*a*_m_ · *D*), (15)

ρ = (8 · *M*)/(*N*_a_ · *a*_m_^3^). (16)

Here *M* is the molar mass of ferrite, g/mol; *N*_a_ = 6.02 10^23^ mol^−1^ is Avogadro’s number.

The anion–cation distances in the tetrahedral (*L*_A_) and octahedral (*L*_B_) coordination of the spinel grid were determined using Formulas (17) and (18):

(17) $$L_{a}=\sqrt{0.1875\cdot a_{m}^{2}},$$

(18) $$L_{B}=\sqrt{0.125\cdot a_{m}^{2}}$$

Ultrastructural images of the samples were obtained on a Quattro S SEM scanning microscope (Thermo Fisher Scientific, Waltham, MA, USA) in the bright-field mode at an accelerating voltage of 100 kV, magnification ×350. FTIR spectroscopy was performed on the Spectrum Two hardware complex (Perkin-Elmer, Shelton, CT, USA).

The catalytic activity was studied using a model solution of an organic dye of methyl orange (λ_max_ = 465 nm). During the experiment, 10 mg of the catalyst was weighed out and placed in a reaction vessel. Then, 10 mL of an organic dye solution with a concentration of 0.05 g/L and 10 mL of a hydrogen peroxide solution with a concentration of 3% were added, and sulfuric acid and sodium alkali solutions with a concentration of 1 mol/L were used to create a certain acidity of the medium. The dye in the solution was analyzed photocolorimetrically using a KFK-2-UHL 4.2 device (Zagorsky Optical and Mechanical Plant, Sergiev Posad, Russia) at certain intervals. A 100 W JC halogen lamp (Camelion, Camelion International Ltd., Shenzhen, China) was used as a light source. The distance from the light source to the surface of the reaction system was 50 mm. Before the experiment, the reaction system was thoroughly mixed while isolated from light for 0.5 h to achieve adsorption/desorption equilibrium.

The degree of destruction P, %, was calculated using Formula (19):

*P* = (*C*_0_ − *C*_t_) · 100/C_0_, (19)

where *C*_0_ is the initial concentration of the dye in the solution, g/L; Ct is the amount of dye that has undergone degradation at the current time, g/L.

## 4. Conclusions

In this work, for the first time, composite organo-inorganic materials containing sunflower husk biochar and nickel(II)-copper(II) ferrites of the composition of Cu_x_Ni_1−x_Fe_2_O_4_ (x = 0.0; 0.5; 1.0) were obtained.

The change in the structural parameters of the inorganic part of the composite material as the chemical composition changed was analyzed. When copper (II) cations were replaced by nickel (II) cations, the average parameter values and the unit cell volume and the cation–anion distance in the octahedral spinel grid were found to change with regularity. The obtained experimental facts can be associated with a change in the ionic radius of the bivalent cation in the spinel grid and the Jahn–Teller effect manifestation.

In the mixed nickel (II)-copper (II) ferrite, an increased concentration of crystal grid defects is noted, caused by a high degree of ferrite inversion and the Jahn–Teller effect manifestation.

The composite materials have a core–shell structure, while the oxide materials form a film on the surface of biochar, repeating its porous structure. This can be useful for the catalytic properties of the materials, as materials with a developed surface are formed, which is important for catalysts.

The obtained materials exhibit high catalytic activity in the reaction of methyl orange decomposition under hydrogen peroxide in an acidic medium. So, in the presence of composites of the composition Cu_0.5_Ni_0.5_Fe_2_O_4_/biochar, NiFe_2_O_4_/biochar, and CuFe_2_O_4_/biochar, it is possible to carry out complete degradation of the organic azo dye in an aqueous solution for 30, 90, and 140 min, respectively. This result can be associated with the formation of the Fenton system during the oxidation–reduction process.

A significant increase in the reaction rate in the system containing mixed nickel–copper ferrite as a catalyst may be associated with the formation of a more defective structure due to the Jahn–Teller effect manifestation, which creates additional active centers on the catalyst surface.

## Figures and Tables

**Table 1:** Catalytic activity of oxide materials for removal of methyl orange.

No.	The Catalyst	Concentration of the Pollutant	Conditions of Catalysis, Degree of Purification	Reference
1	1% Sn-ZnO-graphene	5 mmol/L	UV, 6 h, 89% Visible, 3 h, 98%	[28]
2	5% Sn-ZnO-graphene	5 mmol/L	UV, 6 h, 80.4% Visible, 3 h, 99%	[28]
3	ZnO-Zn_2_TiO_4_	10 mg/L	UV, 3 h, 90%	[29]
4	ZnO(10%)/TiO	10.3 mg/L	Visible, 280 min, 78%	[30]
5	citrate-modified clay	50 mg/L	UV, 90 min, 100%	[31]
6	Fe^2+^, β- Cyclodextrin	26 mkmol/L	Visible, 30 min, 100%	[32]
7	NiFe_2_O_4_/CoMoS_4_	10 mg/L	Visible, 60 min, 99%	[33]
8	Fe_3_O_4_/biochar made from corn cob	25 mg/L	Visible, 14 min, 99.7%	[34]
9	fibrous silica/Fe_3_O_4_	10 mg/L	Visible, 180 min, 80%	[35]

**Table 2:** Structural parameters of spinels.

Sample	*a*	*c*	*c/a*	*a*_m_	*V*	*D*	*L*_A_	*L*_B_
CF	0.8371	0.8598	1.03	0.8446	0.602	109	0.3657	0.2986
CNF	0.8340	0.8732	1.04	0.8351	0.582	130	0.3616	0.2953
NF	0.8341	-		0.8341	0.580	107	0.3612	0.2949

**Table 3:** Characteristics of spinel crystals.

Sample	λ	Formula	ρ, g/sm^3^	δ·10^3^	ε·10^3^
CF	0.24	Fe_0.24_Cu_0.76_[Cu_0.24_Fe_1.76_]O_4_	5.29	0.138	8.47
CNF	0.87	Cu_0.13_Fe_0.87_[Cu_0.37_Ni_0.5_Fe_1.13_]O_4_	5.42	0.144	10.42
NF	0.95	Fe_0.95_Ni_0.05_[Ni_0.95_Fe_1.05_]O_4_	5.38	0.118	7.03

## Data Availability

The original contributions presented in this study are included in the article.
